# An observational cohort study of health outcomes and costs associated with early pregnancy assessment units in the UK

**DOI:** 10.1186/s12913-022-07709-9

**Published:** 2022-03-09

**Authors:** Edna Keeney, Maria Memtsa, Venetia Goodhart, Davor Jurkovic, Gareth Ambler, Nazim Khan, Jeff Round

**Affiliations:** 1 Health Economics Bristol, Population Health Sciences, University of Bristol, Bristol, UK; 2 Elizabeth Garrett Anderson Institute for Women’s Health, University College London, London, UK; 3 Department of Statistical Science, University College London, London, UK; 4 Modelling and Analytical Systems Solutions Ltd, Edinburgh, UK; 5 Institute of Health Economics, Edmonton, Alberta Canada; 6 Faculty of Medicine and Dentistry, Department of Pediatrics, University of Alberta, Edmonton, Alberta Canada

**Keywords:** Early pregnancy, Cohort study, Cost-effectiveness, Quality of life

## Abstract

**Background:**

The objective of this study was to assess the impact of consultant presence, volume of patients seen and weekend opening on the health and cost-related outcomes associated with different Early Pregnancy Assessment Unit (EPAU) configurations.

**Methods:**

This was an observational study with a prospective cohort design. Six thousand six hundred six pregnant women (16 years of age and over) attending EPAUs because of suspected early pregnancy complications were recruited from 44 EPAUs across the UK. The main outcome measures were quality of life, costs, and anxiety.

**Results:**

Costs, quality of life and anxiety scores were similar across configurations with little evidence to suggest an impact of consultant presence, weekend opening or volume of patients seen. Mean overall costs varied from £92 (95% CI £85 - £98) for a diagnosis of normally developing pregnancy to £1793 (95% CI £1346 - £2240) for a molar pregnancy. EQ-5D-5L score increased from 0.85 (95% CI 0.84–0.86) at baseline to 0.91 (95% CI 0.90–0.92) at 4 weeks for the 573 women who completed questionnaires at both time points, largely due to improvements in the pain/discomfort and anxiety/depression dimensions. 78% of women reported a decrease in their anxiety score immediately following their EPAU appointment.

**Conclusions:**

EPAU configuration, as specified in this study, had limited impact on any of the outcomes examined. However, it is clear that care provided in the EPAU has a positive overall effect on women’s health and emotional wellbeing, with significant improvements in EQ-5D and anxiety shown following an EPAU visit.

**Supplementary Information:**

The online version contains supplementary material available at 10.1186/s12913-022-07709-9.

## Background

Complications in early pregnancy are common and account for the largest proportion of emergency work performed in gynaecology departments across the UK [1]. ‘Early pregnancy complications’ is a broad term that encompasses all types of pregnancy loss in the first 12 weeks of gestation (miscarriage, ectopic pregnancy, trophoblastic disease), as well as maternal complications such as hyperemesis gravidarum. Indicatively, it is estimated that, in the UK, per annum, there are 1,000,000 pregnancies, of which, at least 200,000 result in a miscarriage, and, at least 10,000 in an ectopic pregnancy (2007 CEMACH).

In the UK, women with suspected early pregnancy complications are mostly cared for in Early Pregnancy Assessment Units (EPAUs), specialist organisational structures, unique to the National Health Service (NHS). EPAUs aim to provide a dedicated, centralised outpatient service which includes clinical assessment, ultrasound, and laboratory investigations in an attempt to streamline and improve the quality of care for women that experience pain and/or bleeding in early pregnancy.

The number of EPAUs in NHS hospitals has increased exponentially since 1991, when Bigrigg and Read [2] first reported that the introduction of an EPAU at their local hospital resulted in improved quality of care and cost savings. EPAUs are reportedly associated with shortening of the time taken to reach the correct diagnosis as well as a reduction in the number of hospital admissions for women with suspected early pregnancy complications [2]. According to the Association of Early Pregnancy Units (AEPU), there are currently over 200 operational EPAUs in acute NHS hospital trusts in the UK [3]. However, the current paucity of evidence regarding how EPAUs should be organised has led to considerable variation amongst units across the country in the levels of access to their services and the levels of care they provide.

As hospital care is the most expensive element of the health service [4], healthcare providers, especially in cost-constrained systems, have to manage resources carefully. Previous studies have shown that inpatient admissions are significantly reduced when consultants are available to review patients in acute clinical settings, such as emergency departments and medical assessment units [5, 6]. Access to ultrasound diagnostic services provided by the EPAU is essential for the safe and effective management of women with early pregnancy complications. As such, if access to the EPAU is limited, it is likely that the number of precautionary admissions, in particular over the weekend, would be increased until potentially harmful early pregnancy complications, such as ectopic pregnancy, can be ruled out. Similarly, it has also been suggested that, for certain patient groups, higher volume leads to better outcomes, possibly due to greater exposure of clinicians to complex cases, which contributes to better collective team experience and learning [5, 6].

To our knowledge, the cost-effectiveness of EPAUs or different EPAU configurations has never been investigated. The latest NICE guideline on Ectopic Pregnancy and Miscarriage (CG 154) [7] recommended research to establish how different models of care within EPAUs might impact on service outcomes, clinical outcomes, and women’s experience of care. Given the considerable variation between different units and the lack of available health economic evidence, we aimed to examine the costs, health gains and cost-effectiveness associated with different EPAU configurations. This information should assist in making evidence-based recommendations about the future configuration of EPAUs in the UK.

## Methods

Data for this health economic evaluation were collected as part of the VESPA study, a UK-wide prospective mixed-methods study on “**V**ariations in the organization of **E**PAUs in the UK and their effects on clinical, **S**ervice and **PA**tient-centred outcomes” [8]. Following a national survey of EPAUs in the UK, units were randomly selected and invited to participate, with an aim to achieve a sample size of 44 units. The random sampling procedure was conducted to achieve an equal distribution of unit characteristics based on the stratification criteria (planned weekly consultant presence (yes/no), the number of patients seen over 1 year, as reported by the clinicians in charge (low volume of < 2500 appointments annually and high volume of ≥2500) and weekend opening (yes/no)). Full details of the recruitment and random sampling procedure are described elsewhere [8].

The recruitment of the units was completed between December 2015 and April 2016. The inclusion criteria for participants were 1) pregnant women (16 years of age and over), b) attending EPAUs because of suspected early pregnancy complications. Women who were haemodynamically unstable or in severe pain were not approached to participate in the study, as they are not routinely seen in an out-patient setting, such as an EPAU. Each participating EPAU was asked to recruit a minimum of 150 consecutively presented women. A total of 6606 women were recruited to the VESPA study. Resource use data was available for 6531 patients. Of the 6606 women recruited, a total of 4217 consented to participate in the Quality of Life questionnaire arm of the study, 414 subsequently withdrew consent, and the remaining 3803 completed the questionnaires.

Eight unique unit configurations were considered and units were divided into the following configurations:

1. Low volume, no consultant presence, no weekend opening (vcw)
2. Low volume, no consultant presence, weekend opening (vcW)
3. Low volume, consultant presence, no weekend opening (vCw)
4. Low volume, consultant presence, weekend opening (vCW)
5. High volume, no consultant presence, no weekend opening (Vcw)
6. High volume, no consultant presence, weekend opening (VcW)
7. High volume, consultant presence, no weekend opening (VCw)
8. High volume, consultant presence, weekend opening (VCW).

### Costs

Data were captured on resource use relating to EPAU visits and the entire care pathway for each of the patients was analysed. Resource use during the visit/s included staff contact time, blood tests ordered, ultrasounds conducted, and admissions for surgery or observation. All members of staff, including administrative and clinical staff, who had contact with women attending the early pregnancy service were asked to record the type of interaction, the start and end time of their interaction, as well as their staff type. This provided an exact salary cost for each patient based on the salary cost of the staff who provided care and assistance to that patient during their EPAU appointment(s) [8].

A complete case approach was used i.e. costs were only estimated if data were available for each aspect of resource use [9]. Costs were analysed adjusting for the stratification variables (consultant presence, weekend opening and volume) as well as for age and final diagnosis. We used a multi-level model to estimate adjusted costs, allowing for clustering at the unit level. Multi-level models have been recommended for use in health economics, as they are able to incorporate the hierarchical structure of data including patients within centres and provide more appropriate estimates of patient and centre-level effects than ordinary least squares models [10].

The unit cost of an ultrasound associated with an EPAU visit was estimated by the finance team at University College London Hospital as £49.21. The cost of a blood test was based on a study by Czoski-Murray et al. [11] and the cost of admissions for surgery or observation were taken from the NHS Reference Costs 2016/17 [12]. Costs were adjusted to the 2016/17 price year using the Personal Social Services Research Unit index [13]. Sources for the costs used in the primary analysis are provided in Supplementary Table 1. No discount rate was applied as all costs were incurred within 1 year.

### Quality of life

Quality of life data was captured using the EQ-5D-5L questionnaire [14] before the consultation at the initial visit and again between two and 6 weeks post-discharge from the EPAU. This validated questionnaire asks patients to score their own health based on five dimensions: mobility, self-care, usual activities, pain/discomfort and anxiety/depression. Each dimension has five levels: no problems, slight problems, moderate problems, severe problems and extreme problems. This selection results in a five-digit number that is converted into a number between 0 (equivalent to death) and 1 (full health) that expresses the patient’s self-reported health at each time point. The replies were then converted to an index score using the value set for England reported by Devlin et al. [15] Index scores were in-turn used to calculate Quality Adjusted Life Years (QALYs) using the area-under-the-curve approach [16].

Anxiety score data were collected using the Visual Analogue Anxiety Scale (VAS-A) [17] prior to clinical assessment for every visit; whether an initial or clinical follow-up visit. Patients also completed the same scale at the end of every visit. Patients were asked to indicate on a horizontal line (with marks going from 0 to 100) how anxious they felt at that moment, with a mark at the extreme left indicating ‘not at all anxious’ and a mark at the extreme right indicating that they were the ‘most anxious they could ever imagine’. Pre- and post- assessment scores were then compared.

### Presentation of results

We calculated mean total costs per patient, by diagnosis and for each configuration. We also looked at change in QALYs and mean change in anxiety pre- and post- consultation. STATA/MP 16.0 was used for all analyses and *p* values ≤0.05 were deemed statistically significant. A probabilistic sensitivity analysis (PSA) was carried out, which reflects uncertainty around the estimates of costs and QALYs [18, 19]. As the probabilistic analysis requires simulated samples from the mean cost and QALY estimates, Monte Carlo simulation was performed within Excel (Microsoft Corporation, Redmond, WA, USA) to obtain 10,000 simulated samples. A gamma distribution was used for costs and a beta distribution for QALYs.

For each configuration, we analysed the expected total QALYs and expected total cost, averaged over the simulation sample, together with 95% confidence intervals (CIs). We also computed net monetary benefit (NMB) for a given willingness-to-pay per QALY, λ, (ceiling ratio) where NMB is defined as:

$$\mathrm{NMB}=\kern0.5em \mathrm{utility}\kern0.5em \ast \kern0.5em \lambda \hbox{-} \mathrm{cost}$$

This converts utilities to a monetary scale, so that the costs and QALYs can be compared directly. Expected NMB is the average net benefit over the simulation samples. For a given willingness-to-pay threshold λ, the optimal configuration is that with the highest expected NMB. We present expected NMB for λ = £20,000 in accordance with NICE guidelines [20].

## Results

### Costs

Of the 6531 women for whom resource use data was collected, complete data including data on a valid diagnosis was available for 6343. The mean total cost per patient was £225 (SD = 537). This varied depending on final diagnosis with the mean total cost in patients with a normally developing pregnancy being £92, in patients with early embryonic demise £473 and in the 12 patients with molar pregnancy £1793 (Table 1).

**Table 1:** Mean total cost by diagnosis (*N* = 6343)

Diagnosis	Mean cost (£)	Standard Deviation	Number of women
Normally developing pregnancy	92	178	3344
Twin pregnancy	94	45	2
Early intra-uterine pregnancy	106	250	812
Other	112	77	3
Complete miscarriage	180	388	685
Not pregnant	238	607	12
Inconclusive scan (Pregnancy of Unknown Location - PUL)	275	621	149
Early embryonic demise	473	814	800
Incomplete miscarriage	694	952	293
Retained products of conception	1005	1177	123
Ectopic pregnancy	1493	950	108
Molar pregnancy	1793	790	12
All women	225	537	6531

Mean predicted total costs by annual patient volume, consultant presence during opening hours, and weekend opening are shown in Table 2. These costs were estimated by a multi-level model allowing for clustering at the unit level and adjusting for age and final diagnosis. Lower volume, no consultant presence and lack of weekend opening were associated with lower costs than their alternatives (*p*-values < 0.01).

**Table 2:** Mean total cost per patient by configuration (*N* = 6340)

	Mean total cost	95% Confidence Interval (£)	Number of women
Volume < 2500	£212	210–214	3181
Volume ≥ 2500	£245	243–247	3159
Consultant presence – No	£224	222–226	3752
Consultant presence -Yes	£235	232–238	2588
Weekend opening – No	£224	221–227	3079
Weekend opening – Yes	£233	230–235	3261

A multi-level model was again used to adjust total cost for final diagnosis, age, yearly volume, consultant presence and hours open at the weekend with results shown in Table 3. No relationship was evident between the unit configuration variables (yearly volume, weekend opening and consultant presence) and total cost. Age and final diagnosis were the only variables showing a statistically significant relationship with total cost (*p*-value < 0.05). For every year increase in age, a roughly £3 increase in total cost is predicted, holding all other variables constant. All final diagnoses other than twin pregnancy, not pregnant, or ‘other’ were associated with higher costs of early pregnancy care than a final diagnosis of normally-developing pregnancy.

**Table 3:** Regression results. Mean total cost per patient adjusted for patient characteristics and final diagnosis (*N* = 6343)

	Coefficient	Standard Error	95% Confidence Interval	*p*-value
Age	2.74	0.97	0.84–4.64	0.005
Normally developing Pregnancy	15.90	18.96	−21.27 – 53.07	0.40
Early Embryonic Demise	373.34	18.56	336.97–409.71	< 0.01
Incomplete Miscarriage	597.60	28.78	541.18–654.01	< 0.01
Retained Products of Conception	891.65	43.03	807.31–975.98	< 0.01
Complete Miscarriage	83.26	19.75	44.56–121.97	< 0.01
Ectopic Pregnancy	1398.97	45.64	1309.52–1488.42	< 0.01
Inconclusive Scan (PUL)	165.68	39.23	88.80–242.57	< 0.01
Molar Pregnancy	1696.73	134.90	1432.32–1961.13	< 0.01
Other	−2.56	269.49	− 530.74 – 525.63	0.99
Twin Pregnancy	−61.68	330.24	− 708.95 – 585.58	0.85
Not Pregnant	142.53	134.92	−121.90 – 406.96	0.29
Yearly volume (> 2500)	0.00	0.01	−0.01 – 0.12	0.81
Weekend opening (Yes)	0.58	1.01	−1.40 – 2.56	0.57
Consultant presence (Yes)	1.42	1.63	−1.76 – 4.61	0.38

### Quality of life

Complete baseline quality of life data were available for 3764 patients but only 573 women completed the questionnaire at both baseline and the 2–6 week follow-up time point. The median number of days between baseline and follow-up was 26. A total of 2173 women completed the baseline questionnaire only with no follow-up recorded. The patients who completed the questionnaire at both baseline and the 2–6 week follow-up time point had a slightly higher baseline EQ-5D of 0.854 (SD 0.132) compared to a baseline score of 0.845 (SD 0.142) in the sample with no follow up. They were slightly older than the sample with no follow up with a mean age of 32.4 (95% CI 31.9–32.9) compared to 29.1 (95% CI 28.8–29.3). They were also slightly less likely to have had a diagnosis of normally developing pregnancy (324/572 (56.74%) compared to 1262/2173 (58.08%)). The range of diagnoses in both groups is shown in Table 4.

**Table 4:** Range of diagnoses in women with baseline and follow-up data

Diagnosis	% in those with only baseline data	N^a^	% in those with both baseline and follow up data	N^a^
Early intra-uterine pregnancy	13.16	286	12.61	72
Normally developing pregnancy	58.08	1262	56.74	324
Early embryonic demise	10.12	220	12.61	72
Incomplete miscarriage	3.41	74	4.73	27
Retained products of conception	1.33	29	1.05	6
Complete miscarriage	9.99	217	10.86	62
Ectopic pregnancy	1.7	37	0.35	2
Inconclusive scan (PUL)	1.98	43	0.88	5
Molar pregnancy	0.18	4	0.18	1
Other	0.05	1	0	0
Not pregnant	13.16	286	0	0

^a^*N* Number of women with diagnosis

The mean score at baseline for patients with both baseline and 4-week questionnaires returned (573 women) was 0.854 (SD = 0.13) and at an average follow up of 26 days, was 0.91 (SD = 0.11). Table 5 demonstrates the similar increase in scores across diagnoses.

**Table 5:** Mean baseline and follow-up quality of life scores by diagnosis

	Baseline index score	Standard Deviation	Index score at 4 weeks	Standard Deviation	N^a^
Early intra-uterine pregnancy	0.853	0.16	0.904	0.12	72
Normally developing pregnancy	0.859	0.13	0.905	0.11	324
Early embryonic demise	0.822	0.14	0.927	0.09	72
Incomplete miscarriage	0.887	0.08	0.914	0.08	27
Retained products of conception	0.829	0.12	0.869	0.12	6
Complete miscarriage	0.857	0.13	0.920	0.09	62
Ectopic pregnancy	0.930	0.10	0.930	0.10	2
Inconclusive scan (PUL)	0.852	0.06	0.987	0.03	5
Molar pregnancy	0.715	–	0.896	–	1

^a^*N* Number of women with diagnosis

Table 6 shows baseline and follow-up index scores by configuration along with the mean index score, the SD of the mean values over patients, the QALY change (mean index score * (4/52 weeks)) and the number of women who completed the questionnaire at both time points in each configuration. The biggest QALY change was seen in configuration Vcw (high volume, no consultant presence, no weekend opening), although this was based on questionnaires from only 12 women, and the smallest in configuration VcW (high volume, no consultant presence, weekend opening).

**Table 6:** QALYs at 4 weeks by configuration (*N* = 573)

Configuration^a^	Baseline index score	Index score at 4 weeks	Mean index score	Standard Deviation	QALY change	Number of women
vcw	0.871	0.909	0.890	0.09	0.068	112
vcW	0.882	0.926	0.904	0.07	0.070	58
vCw	0.883	0.907	0.895	0.11	0.069	76
vCW	0.871	0.915	0.893	0.09	0.069	52
Vcw	0.907	0.950	0.929	0.09	0.071	12
VcW	0.799	0.898	0.849	0.13	0.065	113
VCw	0.838	0.912	0.875	0.09	0.067	56
VCW	0.849	0.907	0.878	0.09	0.068	94

^a^*v* low volume, *V* high volume, *c* no consultant presence, *C* consultant presence, *w* no weekend opening, *W* weekend opening

### Percentages of people reporting problems at baseline and 4-weeks

The percentage of patients reporting each level of problem on each dimension of the EQ-5D-5L at baseline and 4-weeks was also explored. Table 7 shows that the positive change in patient’s overall health at 4-weeks was largely due to less people reporting pain/discomfort and anxiety/depression. Little change was seen in the percentages of women reporting problems with mobility, self-care or usual activities at baseline and 2 weeks (Table 8).

**Table 7:** Percentage of patients reporting each level of problem with pain/discomfort and anxiety/depression at baseline and 4 weeks

	Pain/discomfort		Anxiety/depression	
Level	Baseline (%)	2 weeks (%)	Baseline (%)	2 weeks (%)
No	42	60	32	55
Slight	41	34	34	32
Moderate	15	4	23	11
Severe	2	1	7	1
Extreme	0	0	3	1
Reporting some problems	58	40	68	46

**Table 8:** Percentage of patients reporting each level of problem with mobility, self-care and usual activities at baseline and 4 weeks (*N* = 573)

	Mobility		Self-care		Usual activities	
Level	Baseline (%)	2 weeks (%)	Baseline (%)	2 weeks (%)	Baseline (%)	2 weeks (%)
No problems	93	93	98	98	78	80
Slight problems	5	6	2	1	17	16
Moderate problems	1	1	0	0	4	3
Severe problems	1	0	0	0	1	1
Unable	0	0	0	0	1	1
Reporting some problems	7	7	2	2	22	20

### Cost per QALY at 4 weeks

The expected total costs and expected total QALYs at 4 weeks for each configuration are shown in Table 9, along with their 95% CIs, estimated from the probabilistic analysis. The table shows that the units with low volume, consultant presence, and no weekend opening (vCw) or low volume, consultant presence and weekend opening (vCW) had the lowest expected costs. The highest costs were found for units with high volume, consultant presence and no weekend opening (VCw). Configurations with high volume, no consultant presence and no weekend opening (Vcw) had the highest expected QALY change at 4 weeks followed by units with low volume, no consultant presence and weekend opening (vcW). Configurations wigh high volume, no consultant presence and weekend opening (VcW) had the lowest expected QALY change. The minimal difference in expected QALYs between configuration types (0.007 between highest and lowest QALY changes) suggests that the configuration types could be assumed to be equivalent in terms of benefit offered on the EQ-5D scale. In this case a comparison between them should effectively be based on minimising total costs. In addition, the CIs show that there is a high degree of uncertainty in the QALY change estimates. The expected net benefit at a £20,000 willingness-to-pay threshold is highest for configurations with the lowest cost (vCw) (£1203) and lowest for configurations with the highest cost (VCw) (£1064).

**Table 9:** Expected total costs, expected total utilities, and expected net benefit at a £20,000 willingness-to-pay threshold with a 4-week timeframe. V refers to yearly volume of patients seen </> 2500, C refers to consultant presence Y/N and W refers to weekend opening Y/N

Configuration^a^	Mean cost (£)	95% Confidence Interval	Mean QALY change	95% Confidence Interval	Probabilistic Net Monetary Benefit	95% Confidence Interval
vCw	173	143–206	0.069	0.053–0.086	1203	882–1556
vCW	180	143–223	0.069	0.055–0.083	1191	915–1496
vcW	216	191–243	0.070	0.059–0.081	1176	956–1409
vcw	226	183–277	0.069	0.055–0.083	1146	873–1440
VcW	227	198–257	0.065	0.047–0.087	1079	705–1508
Vcw	240	180–308	0.072	0.059–0.085	1190	935–1463
VCW	258	224–296	0.068	0.055–0.081	1092	834–1373
VCw	280	232–332	0.067	0.055–0.081	1064	812–1345

^a^*v* low volume, *V* high volume, *c* no consultant presence, *C* consultant presence, *w* no weekend opening, *W* weekend opening

### Pre- and post – consultation anxiety

**Fig. 1:**
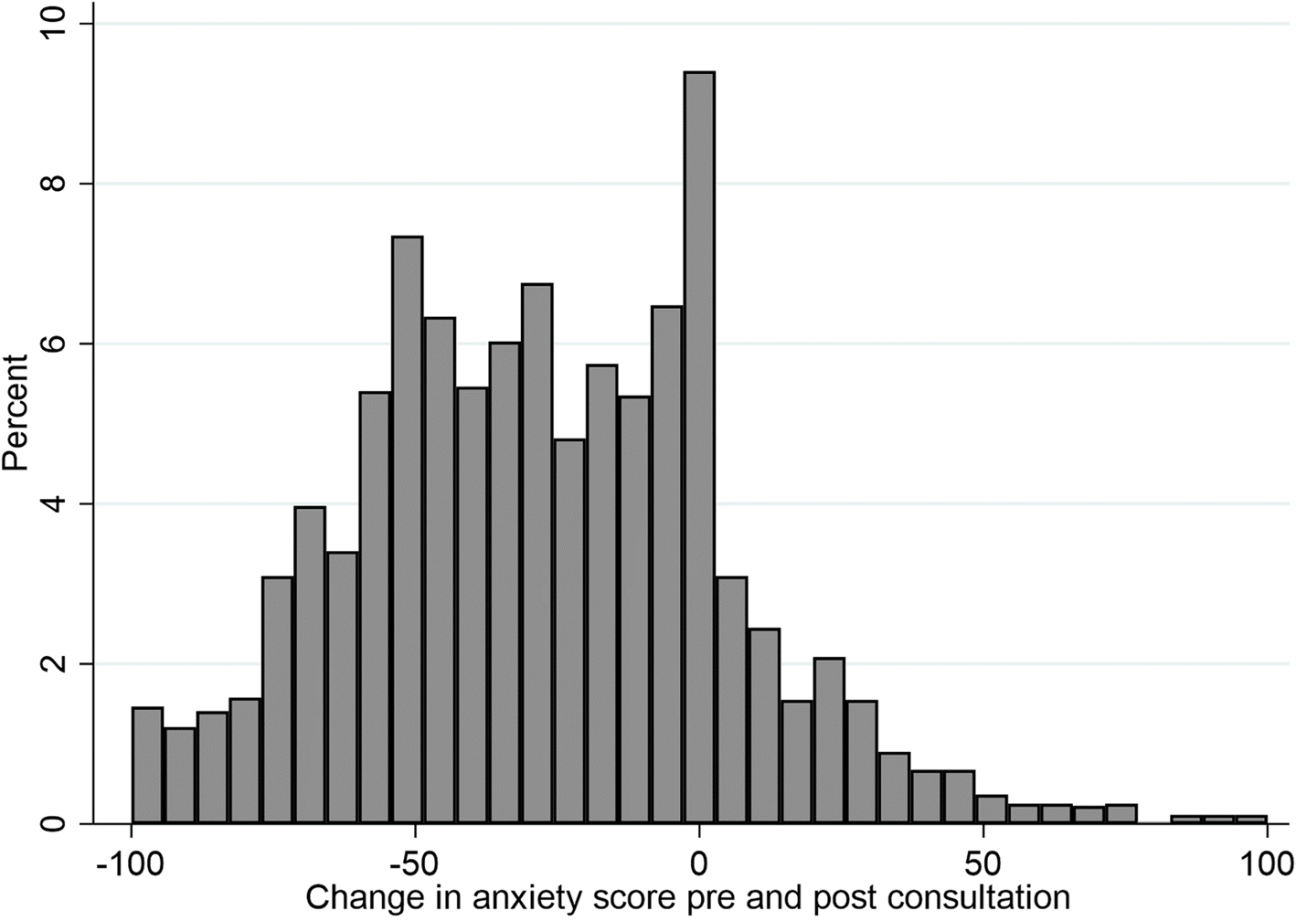
Change in anxiety pre and post consultation

Patients were asked to report their anxiety pre- and post- consultation for every visit at the EPAU. Three thousand five hundred fifty pre- and post- consultation anxiety scores were available. Figure 1 shows that most people (78%) experienced a decrease in their anxiety following their EPAU appointment.

The mean anxiety score pre- and post- consultation and the mean change in anxiety for each configuration are shown in Table 10. vCw had the biggest mean decrease in anxiety whereas vcW had the smallest, although differences were minimal.

**Table 10:** Change in anxiety pre and post consultation

Configur-ation^a^	Mean anxiety score pre-consultation	95% Confidence Interval	Mean anxiety score post-consultation	95% Confidence Interval	Mean change in anxiety pre and post consultation	95% Confidence Interval
vcw	56	(54–58)	27	(25–29)	− 29	(−32, − 27)
vcW	54	(51–57)	31	(28–34)	−23	(− 27, − 20)
vCw	56	(53–59)	26	(24–29)	−30	(− 33, − 27)
vCW	57	(54–61)	30	(26–33)	−28	(− 32, − 24)
Vcw	56	(52–61)	27	(23–32)	− 29	(− 34, − 24)
VcW	56	(54–58)	29	(27–32)	− 27	(−29, − 24)
VCw	59	(56–62)	34	(30–38)	− 25	(− 28, − 21)
VCW	59	(57–61)	31	(28–33)	−28	(−31, − 25)

^a^*v* low volume, *V* high volume, *c* no consultant presence, *C* consultant presence, *w* no weekend opening, *W* weekend opening

## Discussion

It is evident that the total cost per woman attending an EPAU with early pregnancy complications in terms of blood tests, ultrasounds, admissions and staff costs is, as expected, strongly dependent on the final diagnosis, and to a lesser degree the woman’s age. Over 60% of women recruited in this study had a diagnosis of normally developing or early intra-uterine pregnancy with a mean cost of £92 and £106 respectively. Women with higher service use costs were those experiencing miscarriage or molar pregnancy (£180 and £1793).

Mean total cost by configuration varied from £189 - £257 per patient when adjusted for age and final diagnosis. Patient selection was not based on the severity of their condition; all women who consecutively presented to the participating EPAUs were invited to participate in the study prior to establishing their diagnosis. On the basis of our pilot work, significant differences in patient condition across the different units were not anticipated [8]. The % or pregnancies that were ‘normal’ varied between 50.3 and 76.3% across all units, with a mean of 68.9%. The lowest average cost per patient was encountered in small volume units with consultant presence that were closed over the weekend, although, overall, there was little evidence to suggest that the differences in cost were related to consultant presence, volume of patients seen or weekend opening. The findings suggest that individual unit factors or the characteristics of women seen at different units drive increased admissions, and therefore differences in costs; however, given the limitations of the study design, it cannot be conclusively determined that unit configuration does not play a role.

Women’s overall health at 4 weeks measured on the EQ-5D-5L scale improved with the average score increasing from 0.854 at baseline to 0.91 at a mean follow up of 26 days for the 573 women who completed questionnaires at both time points. This was mainly due to the fact that considerably fewer women reported problems in the pain/discomfort and anxiety/depression dimensions. Only minimal variation in mean QALY change was witnessed between configurations (0.065–0.071).

The probabilistic cost-effectiveness analysis showed that small volume units with consultant presence that were closed over the weekend had the highest expected net benefit (ENB) at a £20,000 willingness-to-pay threshold at 4 weeks (£1203). On the other end of the spectrum, small volume units without consultant presence that were open at the weekends had the lowest ENB (£1064). However, due to uncertainty, it is not possible from this data to conclusively recommend a particular EPAU configuration.

The results on the Visual Analogue Anxiety Scale showed that 78% of 3550 women experienced a decrease in their anxiety immediately following their EPAU appointment. A variation of 23 to 30 points on the anxiety scale across configuration types was found. The largest decrease in anxiety was shown in women who attended small volume units with consultant presence and no weekend opening.

One limitation of the study is that we were unable to conduct a randomised controlled study (to compare hospitals with an EPAU against hospitals without an EPAU) as in the vast majority of NHS hospitals, an EPAU is operational. It was, therefore, not possible to observe what may be a considerable number of confounding factors which may influence on mean costs and health gains. As it was not possible to conduct such a trial, the unit characteristics (presence of consultants, weekend opening hours and volume of patients seen) were selected as variables on which to compare since they were believed, a priori, to be likely to impact on the costs and outcomes of women being treated. It was also necessary to categorise units based on a certain set of characteristics to make it possible to select a sample of them with enough variation to be meaningfully different. However, as the number of characteristics increases, so too does the number of units and patients required to detect any effect attributable to those characteristics. In order to keep the sample size feasible, we were therefore required to limit the number of characteristics to three.

A further limitation is the poor response rate observed at the four-week follow up period. Although best practice for maximising response rates was followed, obtaining the target number of observations at each time point required sending a large number of questionnaires. In addition, many questionnaires were returned late. For the four-week follow-up analysis, we used any questionnaire that was returned between two and 6 weeks post visit. While this approach is not optimal compared to a more positive response rate being achieved, we believe it is more robust than using only questionnaires returned at 4 weeks. We believe our approach captures information on quality of life at a time point relevant to the follow-up time periods. It was also reassuring to find that the group that completed the questionnaire within this timeframe was representative in terms of diagnoses (the largest predictor of costs and outcomes).

## Conclusions

Our study has shown that EPAU configuration, as specified in this study, had limited impact on costs, anxiety or health outcomes measured as QALYs although this may be due to issues with the configuration allocation. As we were unable to compare hospitals with an EPAU against hospitals without an EPAU, it is difficult to form a conclusion on whether EPAUs are cost-effective in general. However, overall, it is clear that care provided in the EPAU had a positive effect on women’s health and emotional wellbeing with three quarters of women reporting a decrease in anxiety scores and a positive change in their overall health at 4 weeks.

## Supplementary Information

**Additional file 1.**

## Data Availability

The datasets used and/or analysed during the current study are available from the corresponding author on reasonable request.
