# Supplementary material for: An observational cohort study of health outcomes and costs associated with early pregnancy assessment units in the UK
Source: BMC Health Serv Res. 2022 Mar 9;22:319. doi: 10.1186/s12913-022-07709-9 (PMC8905996; doi:10.1186/s12913-022-07709-9)
Supplement: Supplementary file 1 — Additional file 1. [file 12913_2022_7709_MOESM1_ESM.docx]

# SUPPLEMENTARY TABLES

### Supplementary Table 1. Unit costs for tests and admissions

|  | Cost (£) | Source |
| --- | --- | --- |
| Ultrasound | 49.21 | Unit cost of Ultrasounds (excluding Medical Staffing) charged to activity with HRG MA36Z at UCL Hospitals NHS Foundations Trust |
| Blood test | 6 | Czoski-Murray et al^30^ Cost of a full blood count test. |
| Admission for emergency/semi-elective surgery | 2,035 | NHS reference costs 2016/17^31^ Admission for threatened or spontaneous miscarriage with interventions. MB08A Non-elective inpatient (NEL) |
| Admission for observation only | 1,641 | NHS reference costs 2016/17^31^ Admission for threatened or spontaneous miscarriage without interventions. MB09A NEL |

Supplementary Table 2. Summary of data collection tools, sources and timings

| **Data strand** | **Data collection tool** | **Data source** | **When collected** |
| --- | --- | --- | --- |
| Clinical outcomes in EPAU | Study CRF | All eligible women attending the EPAU service | At site initiation until data for 150 women had been collected. |
| Emergency hospital care audit | Routine hospital data | All pregnant women <14 weeks gestation attending emergency hospital services and admitted to hospital | Retrospective for 3 months after clinical EPAU data completion at each participating site |
| Patient satisfaction | SAPS Q  Modified Newcastle-Farnworth Q  LMUP | Women attending the service who consented to the questionnaire arm of the study | Planned at 2 weeks post-discharge |
| Staff satisfaction | Modified NHS survey | Staff providing care to women who attended the early pregnancy service | During patient recruitment |
| Qualitative interviews | Telephone interviews based on the agreed topic guide | Women attending the service who consented to be interviewed | Following analysis of clinical outcomes & SAPS Q |
| Health economic evaluation | EQ-5D 5L Q  VAS-A  VAS-A  EQ-5D 5L Q  CSRI Q  EQ-5D 5L Q | Women attending the EPAU service who consented to the questionnaire arm of the study | Upon arrival at EPAU and before consultation at initial visit  Following initial visit  At any clinical follow-up visit, prior to and following clinical assessment  2 weeks post-discharge  3 months post-discharge |
| Workforce analysis | Study CRF | Any interaction with any member of EPAU staff that had contact with women attending the service | During patient recruitment |

(*CRF: Case Report Form, SAPS: Short Assessment of Patient Satisfaction, LMUP: London Measure of Unplanned Pregnancy, EQ-5D-5L: EuroQol-5D 5L questionnaire, VAS-A: VAS-A: Visual Analogue Anxiety Scale, CSRI Q: Client Service Receipt Inventory)
